# Functional Analysis of a Putative Type III Secretion System in Stress Adaption by *Mesorhizobium alhagi* CCNWXJ12-2^T^

**DOI:** 10.3389/fmicb.2018.00263

**Published:** 2018-02-20

**Authors:** Xiaodong Liu, Yantao Luo, Zhefei Li, Gehong Wei

**Affiliations:** State Key Laboratory of Crop Stress Biology in Arid Areas, Shaanxi Key Laboratory of Agricultural and Environmental Microbiology, College of Life Sciences, Northwest Agriculture & Forestry University, Yangling, China

**Keywords:** *Mesorhizobium alhagi*, type III secretion system, stress adaption, β-galactosidase assay, Na^+^ content measurement

## Abstract

*Mesorhizobium alhagi* CCNWXJ12-2^T^, isolated from root nodules of the desert plant *Alhagi sparsifolia*, contains two type III secretion systems (T3SSs). T3SSs are specialized machinery with wide distribution in bacteria that inject effector proteins into target cells. Our previous study showed that the expression of *M. alhagi* T3SS1 is upregulated in high-salt conditions. Here, phylogenetic analysis of T3SS1 using the core protein RhcU suggested that T3SS1 belongs to the α-Rhc II subgroup of the Rhc T3SS family. To elaborate the function of *M. alhagi* CCNWXJ12-2^T^ T3SS1 in stress adaption, two T3SS1 mutants (*ΔrhcQ* and *ΔMA29250*) were constructed and analyzed. β-galactosidase transcriptional fusion assays showed that activity of the promoter of T3SS1 was induced by salts. Mutant *ΔrhcQ* was more sensitive to NaCl and LiCl than the wild-type, but *ΔMA29250* was not. Both mutants were more sensitive to KCl than the wild-type. The intracellular Na^+^ concentration in *ΔrhcQ* in high-NaCl conditions (0.4 M) increased by 37% compared to that of the wild-type strain, while the Na^+^ concentration in *ΔMA29250* increased by 13%. The K^+^ concentration in both mutants increased by 16% compared to the wild-type in high-KCl conditions (0.3 M). Strain *ΔrhcQ* showed decreased survival compared to the wild-type after treatment with H_2_O_2_, while the survival rate of *ΔMA29250* was almost the same as that of the wild-type. Antioxidant enzyme activities in *ΔrhcQ* were lower than those in the wild-type strain, but this was not the case for *ΔMA29250*. Our data elucidate the beneficial effects of T3SS1 in the adaption of *M. alhagi* CCNWXJ12-2^T^ to stress.

## Introduction

The type III secretion systems (T3SSs) are widespread in Gram-negative bacteria; their main function is to translocate effector proteins from bacteria into host cells. Many reports have shown that T3SSs play an important role in bacterial virulence (Galán and Collmer, 1999; Rolhion et al., 2016; Wang et al., 2016) and in symbiotic interactions between microorganisms and their hosts (Silver et al., 2007; Buttner and He, 2009; Correa et al., 2012). Because of the important roles that T3SSs play in bacteria–host cell interactions, the T3SSs are arguably one of the best characterized protein injection systems (Galán et al., 2014). There are also some reports on T3SS expression in high-salt conditions. In *Yersinia enterocolitica*, the expression of a chromosomally encoded T3SS was found to be triggered in high-salt conditions and at low temperature (Haller et al., 2000). Microarray analysis of *Pseudomonas aeruginosa* showed that expression of T3SS and associated cytotoxins was upregulated in hyperosmotic stress conditions (Aspedon et al., 2006). Similar results were also found for *Salmonella enterica* serovar Typhimurium (Mizusaki et al., 2008). Most research has focused on changes in pathogenicity and protein secretion between T3SS-related mutants and wild-type strains (Haller et al., 2000; Galán et al., 2014). However, little is known about the functions of T3SSs in stress resistance.

In rhizobial T3SSs, the core genes are called the *rhc* genes (Rhizobium conserved) to distinguish them from phytopathogenic bacterial T3SSs (Viprey et al., 1998). Phylogenetic analysis of T3SS core genes suggested that the rhizobial T3SSs could constitute an individual family (the Rhc family); further analysis showed that the Rhc family could be divided into four subgroups: α-Rhc I, α-Rhc II, α-Rhc III, and β-Rhc (Tampakaki, 2014). Most T3SSs in the α-Rhc I group play an important role in symbiosis formation, for example, in *Bradyrhizobium japonicum* (Krause et al., 2002), *Sinorhizobium fredii* (Lorio et al., 2004), and *Mesorhizobium loti* (Sanchez et al., 2012). However, there is little knowledge regarding the functions of T3SSs in group α-Rhc II (Tampakaki, 2014).

*Mesorhizobium alhagi* CCNWXJ12-2^T^ (=ACCC 14561^T^ = HAMBI 3019^T^) is a soil-dwelling α-proteobacterium isolated from root nodules of the desert plant *Alhagi sparsifolia* and possessing high salt resistance (up to 0.8 M NaCl) (Chen et al., 2010). Two T3SSs, designated as T3SS1 and T3SS2, were found in the draft genome sequence of *M. alhagi* CCNWXJ12-2^T^ (GenBank accession number AHAM00000000) (Zhou et al., 2012). To investigate the transcriptional changes in *M. alhagi* CCNWXJ12-2^T^ in high salt conditions, a transcriptome analysis was conducted previously and the results revealed that the expression of T3SS1 was upregulated significantly in high-salt conditions, while the expression of T3SS2 was downregulated (GEO accession number GSE57306) (Liu et al., 2014). Phylogenetic analysis of RhcU (or SctU), a core protein of T3SS, suggested that *M. alhagi* T3SS1 belongs to the α-Rhc II group and T3SS2 belongs to the α-Rhc I group.

The upregulation of *M. alhagi* T3SS1 in high-salt conditions and little knowledge of T3SS functions in α-Rhc II group prompted us to explore here the role of T3SS1 in salt resistance and stress adaption by *M. alhagi* CCNWXJ12-2^T^. The promoter activity of T3SS1 was studied under salt stresses and the phenotypes of two T3SS1 gene knockout strains were compared to the wild-type strain. Our results showed that T3SS1 plays an important role in stress adaption in *M. alhagi* CCNWXJ12-2^T^. We propose a new function of the α-Rhc II group T3SSs in rhizobia.

## Materials and Methods

### Bacterial Strains and Growth Conditions

**Table 1** lists the bacterial strains and plasmids used in this work. The wild-type strain of *M. alhagi* CCNWXJ12-2^T^, *ΔrhcQ* and *ΔMA29250* mutants, and their complemented strains were typically grown in tryptone–yeast extract (TY) broth (5 g tryptone, 3 g yeast extract, and 0.7 g CaCl_2_⋅2H_2_O per liter) at 28°C. Salt mannitol (SM) medium (0.2 g MgSO_4_⋅7H_2_O, 0.1 g CaCl_2_, 0.5g KNO_3_, 0.5 g K_2_HPO_4_, 0.1 g NaCl, 10 g mannitol, 75 mg pantothenic acid, 75 mg biotin, and 75 mg thiamine per liter) was used to isolate gene knockout mutants and complementation strains. *Escherichia coli* was grown in Luria-Bertani (LB) broth (10 g tryptone, 5 g yeast extract, and 10 g NaCl per liter) at 37°C. All bacteria were incubated in aerobic conditions at 180 rpm. Where necessary, antibiotics were added at the following concentrations: kanamycin 100 μg/ml; gentamicin 50 μg/ml.

**Table 1:** Bacterial strains and plasmids used in this study.

Strain or plasmid	Description^a^	Source or reference
*Escherichia coli*		
DH5α	*endA hsdR17 supE44 thi-1 recA1 gyrA relA1 Δ*(*lacZYA-argF*)*U169 deoR* [Φ*80 dlacΔ*(*lacZ*)*M15*]	Hanahan, 1983
MM294	*supE44*^-^ *rfbD1 spoT thi-1 endA1 hsdR17 pro*	Watanabe et al., 1990
DH-TP13	DH5α carrying pBMLTP13	
DH-*rhcQ*	DH5α carrying pK18*rhcQ*	This study
DH-C*rhcQ*	DH5α carrying pBML*rhcQ*	This study
DH-*MA29250*	DH5α carrying pK18*MA29250*	This study
DH-C*MA29250*	DH5α carrying pBML*MA29250*	This study
*M*esorhizobium *alhagi*		
XJ12-2	Wild-type	Chen et al., 2010
XJTP13	XJ12-2 carrying pBMLTP13	This study
*ΔrhcQ*	XJ12-2 *ΔrhcQ*	This study
C*ΔrhcQ*	*ΔrhcQ* carrying pBML*rhcQ*	This study
*ΔMA29250*	XJ12-2 *ΔMA29250*	This study
C*ΔMA29250*	*ΔMA29250* carrying pBML*MA29250*	This study
Plasmids		
pRK2013	Broad-host-range helper vector; Tra^+^ Km^r^	Ditta et al., 1980
pK18*mobsacB*	Suicide vector derived from plasmid pK18; Mob^+^ *sacB* Km^r^	Schafer et al., 1994
pBBR1MCS-5	Broad-host-range cloning vector; Gm^r^	Kovach et al., 1995
pBML	pBBR1MCS-5 containing genes of *LacZ and LacY*	unpublished data
pBMLTP13	pBML containing promoter of T3SS1	This study
pK18*rhcQ*	pK18*mobsacB*::*rchQ*	This study
pBML*rhcQ*	pBML carrying *rhcQ*	This study
pK18*MA29250*	pK18*mobsacB*::*MA29250*	This study
pBML*MA29250*	pBML carrying *MA29250*	This study

*^*a*^Km^*r*^, kanamycin resistance; Gm^*r*^, gentamicin resistance*.

### Phylogenetic Analysis of T3SSs

Phylogenetic analysis was performed using the protein sequences of RhcU, one of the core T3SS proteins, including RhcU1 (locus_tag = “MAXJ12_29220,” from T3SS1) and RhcU2 (locus_tag = “MAXJ12_24267,” from T3SS2). Other closely related RhcU sequences of rhizobia were retrieved from the NCBI^1^ database. MEGA 5.1 software was used to align the protein sequences with the multiple alignment method ClustalW and then to analyze the phylogenetic relationships between different species using the neighbor-joining method with 1000 bootstrap replicates (Tamura et al., 2011). Evolutionary distances were computed using Poisson correction and are expressed in terms of the number of amino acid substitutions per site (Zuckercandl and Pauling, 1965).

### Construction of Promoter Reporter Strain

The promoter reporter plasmid pBML was derived from pBBR1MCS-5. The *lacZ* promoter in pBBR1MCS-5 was deleted and a fragment of *lacZY* cloned from *E. coli* K-12 was used to replace *lacZ*α in pBBR1MCS-5; the new plasmid was called pBML. A DNA fragment (named TP13) containing *rhcC* and the putative promoter of T3SS1 (the upstream 200 bp of *rhcC*) was amplified from *M. alhagi* CCNWXJ12-2^T^ genomic DNA using primers TP1 and TP3 (Supplementary Table S1). The DNA fragment was digested with *Hin*dIII and *Xba*I and then cloned into pBML digested with the same restriction enzymes. Finally, the promoter reporter plasmid, designated pBMLTP13, was transformed into *E. coli* DH5α and the strain was named DH-TP13.

To generate promoter reporter strain *M. alhagi* XJTP13, a triparental mating procedure was conducted as described previously to transform the plasmids from *E. coli* into CCNWXJ12-2^T^ (Liu et al., 2014). Briefly, strain DH-TP13, strain *E. coli* MM249 containing helper plasmid pRK2013, and *M. alhagi* CCNWXJ12-2^T^ were mixed together and incubated on a TY plate for 4 days. Then, SM agar plates containing gentamicin were used to isolate *M. alhagi* containing pBMLTP13. Strain XJTP13 was verified by PCR and sequencing.

### β-Galactosidase Transcriptional Fusion Assay

Strain XJTP13 was first grown to OD_600_ ≈0.8 in 20 ml TY broth containing gentamicin. Then, 20 μl suspensions were added to 20 ml TY broth containing gentamicin and different concentrations of additional salts (0, 0.1 M NaCl, 0.2 M NaCl, 0.3 M NaCl, 0.4 M NaCl, 0.1 M LiCl, 0.2 M LiCl, 0.1 M KCl, 0.2 M KCl, or 0.3 M KCl) and grown to OD_600_ ≈0.8. Then, 100 μl of each culture was subjected to β-galactosidase activity assay using o-nitrophenyl-β-D-galactopyranoside as the substrate (Zhang and Bremer, 1995). Three independent biological repetitions were conducted.

### Construction of Gene Knockout Mutants and Complementation Strains

Supplementary Table S1 lists primers used in construction of gene knockout mutants and complemented strains. A *rhcQ* (locus_tag = “MAXJ12_29275”) in-frame deletion mutant of *M. alhagi* CCNWXJ12-2^T^ was constructed using plasmid pK18*rhcQ*. Primer pairs RhcQ-US/RhcQ-UA and RhcQ-DS/RhcQ-DA were used to amplify the 522-bp upstream DNA fragment and 568-bp downstream DNA fragment of *rhcQ*, respectively. The upstream and downstream DNA fragments were digested with *Eco*RI/*Xba*I and *Xba*I/*Hin*dIII, respectively, then the digested DNA fragments were cloned into linearized plasmid pK18*mobsacB* digested with *Eco*RI and *Hin*dIII, to generate pK18*rhcQ*.

An in-frame deletion mutant of gene *MA29250* (locus_tag = “MAXJ12_29250”) was constructed using plasmid pK18*MA29250*. The procedure used to produce pK18*MA29250* was similar to the procedure for pK18*rhcQ* construction. Briefly, the upstream and downstream DNA fragments of *MA29250* were amplified using primer pairs MA29250-US/MA29250-UA and MA29250-DS/MA29250-DA and then digested using the same restriction enzymes as used for pK18*rhcQ* construction. The digested DNA fragments were then cloned into plasmid pK18*mobsacB* digested with *Eco*RI and *Hin*dIII to generate pK18*MA29250*.

To complement the *ΔrhcQ* and *ΔMA29250* mutants, primer pairs CRhcQA/CRhcQB and CMA29250A/CMA29250B were used to amplify *rhcQ* and *MA29250* gene fragments from genomic DNA of *M. alhagi* CCNWXJ12-2^T^. The gene fragments were then cloned into plasmid pBML digested with *Xba*I using the ClonExpress MultiS One Step Cloning Kit (Vazyme Biotech, China), to generate complementation plasmids pBML*rhcQ* and pBML*MA29250.*

For construction of in-frame deletion mutants and complementation strains, a triparental mating procedure was conducted as described previously (Liu et al., 2014). Briefly, the recipient strains (the wild-type strain or the mutants), the donor strains (*E. coli* DH5α containing the constructed plasmids), and the helper strain (*E. coli* MM249 containing helper plasmid pRK2013) were grown to log phase and mixed together in the ratio 4:1:1. The mixtures were cultured on TY plates for 4 days. For in-frame deletion mutant isolation, SM plates containing kanamycin were used to isolate single exchange mutants (gene knockout plasmids integrated into the genome of *M. alhagi* CCNWXJ12-2^T^), then TY plates containing sucrose (5 g/100 ml) were used to isolate the double exchange mutants (in-frame gene deletion mutants). SM agar plates containing gentamicin were used initially to isolate the complementation strains; the single exchange gene knockout mutants isolated from SM plates were then smeared onto TY plates containing sucrose (5 g/100 ml) to isolate the double exchange mutants. The gene knockout mutants and complemented strains were verified by PCR.

### Salt Resistance Assay

Salt resistance assays were conducted as described previously (Liu et al., 2016). Wild-type *M. alhagi* CCNWXJ12-2^T^, *ΔrhcQ* and *ΔMA29250*, and the complemented strains were grown to log phase in 20 ml TY broth with shaking at 28°C. Next, 20 μl of the cultures were inoculated into 20 ml fresh TY broth and grown to OD_600_ ≈0.8. The cultures were diluted fourfold with sterile H_2_O, then 5 μl of each were spotted onto TY agar plates with or without salts (NaCl 0.4 M, LiCl 0.3 M, or KCl 0.3 M). Plates were incubated at 28°C for 4 days.

### Measurement of Total Cellular Na^+^ and K^+^ Concentrations

Inocula were prepared as described in the section on salt resistance assays. Each inoculum (500 μl, OD_600_ ≈0.2) was smeared onto TY agar plates containing different salts (no added salt, 0.4 M NaCl or 0.3 M KCl) and incubated at 28°C for 4 days (plates containing 0 and 0.4 M NaCl) or 7 days (plates containing 0.3 M KCl). The bacteria were then collected in 1.5-ml sterile tubes using sterile stainless steel spoons and dried at 60°C for 10 h using an air dryer. The total cellular Na^+^ and K^+^ concentrations were measured using an atomic absorption spectrophotometer (Hitachi, Tokyo, Japan).

### Hydrogen Peroxide Sensitivity Assay

H_2_O_2_ resistance assays were conducted as described previously (Liu et al., 2016). Wild-type *M. alhagi* CCNWXJ12-2^T^, *ΔrhcQ, ΔMA29250*, and the complementation strains (C*ΔrhcQ* and C*ΔMA29250*) were cultured to OD_600_ ≈0.6 at 28°C in TY broth containing antibiotics and then diluted with sterile H_2_O to OD_600_ ≈0.1. After treatment with 10 mM H_2_O_2_ for 30 min, the survival rates of all the strains were measured by colony counts compared to cells without peroxide treatment, calculated as follows: [(CFU per ml after treatment with H_2_O_2_)/(CFU per ml before treatment with H_2_O_2_)] × 100.

### Antioxidant Enzyme Activity Assay

A previous report showed that peroxidase activity was not detectable in *M. alhagi* CCNWXJ12-2^T^ (Liu et al., 2016), so here, we measured catalase (CAT) and superoxide dismutase (SOD) activities in normal and stressed conditions. Wild-type *M. alhagi* CCNWXJ12-2^T^, *ΔrhcQ, ΔMA29250*, C*ΔrhcQ*, and C*ΔMA29250* were grown in TY broth containing antibiotics to OD_600_ ≈0.8. Each strain (20 μl suspension) was inoculated into two bottles containing 20 ml TY broth and one bottle containing 20 ml TY broth plus 0.4 M NaCl, and grown to OD_600_ ≈0.8. For the two bottles without additional NaCl, one was treated with 0.1 mM H_2_O_2_ for 30 min and the other without. All cells were collected in 50-ml tubes by centrifugation, resuspended in phosphate-buffered saline, and lysed by ultrasonication. The antioxidant enzyme activities were measured using a CAT Assay Kit and a SOD Assay Kit (Suzhou Comin Biotechnology, China). Total soluble protein concentrations were determined using a BCA Protein Assay Kit (CoWin Biosciences, China).

### Statistical Analysis

Statistical differences between the wild-type strain and the mutants in different conditions were assessed by *t*-test using SPSS version 15.0 (SPSS Inc., Chicago, IL, United States). Differences were considered significant at *P* < 0.05.

## Results

### Phylogenetic Analysis of T3SSs in *M. alhagi* CCNWXJ12-2^T^

**FIGURE 1:**
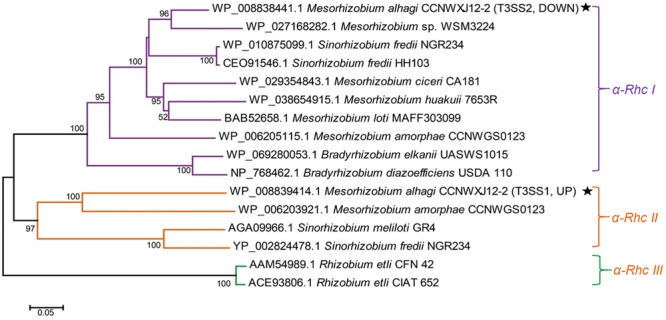
Neighbor-joining tree of RhcU sequences from two type III secretion systems (T3SSs) in the draft genome of *M. alhagi* CCNWXJ12-2^T^. The black stars indicate T3SS1 and T3SS2. UP and DOWN indicate the expression pattern of the T3SSs in high-salt conditions (3). Evolutionary distances were estimated using the *p*-distance method and are in units of the number of amino acid differences per site. Numbers to the left of the branches are bootstrap percentages for 1000 replications. Bootstrap values > 50% are shown, and the scale bar represents the number of substitutions per site. Evolutionary analysis was conducted using MEGA 5.1 (Tamura et al., 2011).

Protein sequences of RhcU, one of the core T3SS proteins, were used to build an evolutionary tree of different rhizobia. Three subgroups of Rhc family T3SSs were apparent in the phylogenetic analysis. *M. alhagi* CCNWXJ12-2^T^ T3SS1 was in subgroup α-Rhc II and T3SS2 was in subgroup α-Rhc I (**Figure 1**).

### T3SS1 Promoter Activity Is Induced by Salts

**FIGURE 2:**
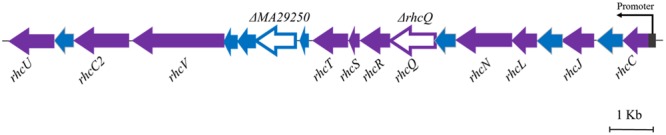
Genetic organization of *M. alhagi* CCNWXJ12-2^T^ T3SS1. Purple arrows indicate conserved T3SS structural component genes. Blue arrows indicate hypothetical protein encoding genes. The genes chosen to construct in-frame deletion mutants are shown as open triangles and the mutant names are above the genes. The putative promoter of T3SS1 is shown as a dark gray rectangle and the arrow indicates the direction of transcription.

**FIGURE 3:**
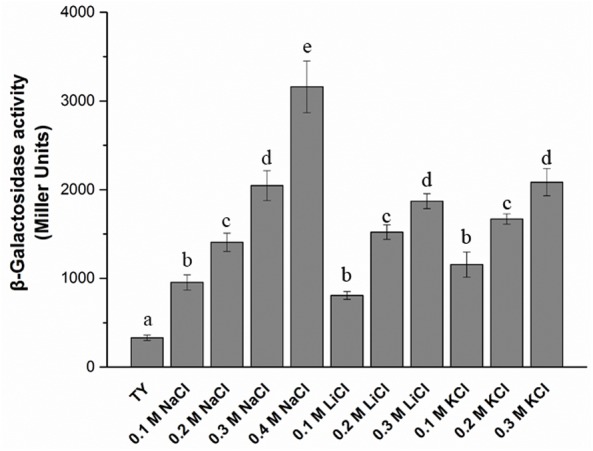
β-galactosidase activity of promoter reporter strain *M. alhagi* XJTP13 on exposure to different salts and salt concentrations. Strain XJTP13 was grown to OD_600_ ≈0.8 in TY broth containing added salts (0–0.4 M NaCl, 0.1–0.2 M LiCl, or 0.1–0.3 M KCl). Then, 100 μl of each culture was taken for β-galactosidase activity assay. Values given are means ± SDs of three replicates. The significance of differences is shown at the *P* < 0.05 level (*t*-test). Different lowercase letters mean significant differences between two columns.

The T3SS1 genes are in an operon in the genome of *M. alhagi* CCNWXJ12-2^T^ (**Figure 2**). The putative promoter (200 bp upstream of *rhcC*) (**Figure 2**) was used with the whole gene of *rhcC* to construct a promoter reporter plasmid. The promoter activities were measured in different conditions using β-galactosidase tests. The β-galactosidase activities increased with increasing salt concentration (**Figure 3**). At the same concentration of different salts (i.e., NaCl, LiCl, and KCl), the β-galactosidase activities were almost the same. The β-galactosidase activities at the lowest salt concentration tested (0.1 M) were almost three times higher than that in the control group (no added salt), while at salt concentrations of 0.3 M they were about six times higher than that in the control group.

### Decrease in Salt Resistance of T3SS1-Related Mutants

**FIGURE 4:**
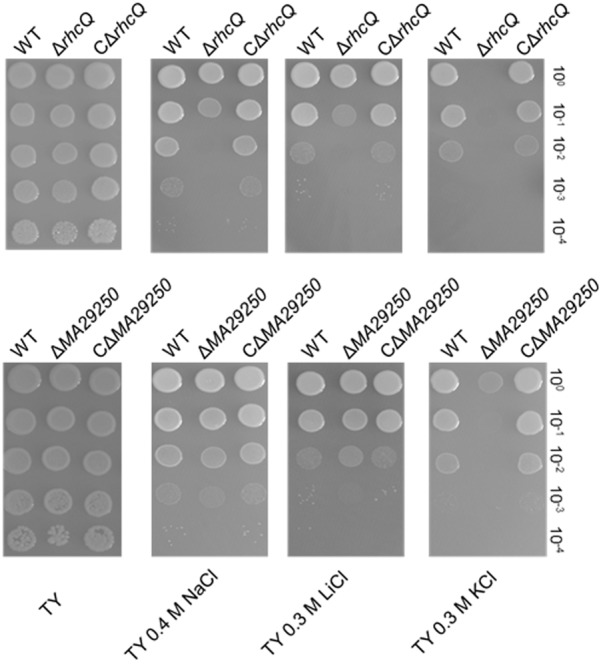
Sensitivity of *M. alhagi* CCNWXJ12-2^T^ to different salts. Wild-type (WT), mutants (*ΔrhcQ* and *ΔMA29250*), and the complementation strains (C*ΔrhcQ* and C*ΔMA29250*) were grown to OD_600_ ≈0.8 in TY broth containing appropriate antibiotics, and then adjusted to OD_600_ ≈0.2. The inocula were serially diluted and spotted onto TY agar plates containing no additional salt or containing 0.4 M NaCl, 0.3 M LiCl, or 0.3 M KCl. The 10-fold serial dilutions are shown. All plates were incubated at 28°C for 4 days.

Two gene knockout mutants, *ΔrhcQ* and *ΔMA29250*, were constructed to study the role T3SS1 plays in salt resistance. One of the genes knocked-out (**Figure 2**) is *rhcQ* (locus_tag = “MAXJ12_29275”), encoding a component of the C-ring of T3SS1 which plays an important role in T3SS assembly, and the other is *MA29250*, encoding a hypothetical protein. *MA29250* (locus_tag = “MAXJ12_29250”) was randomly chosen to study its function. The salt resistance of strain *ΔrhcQ* was decreased compared to the wild-type strain (**Figure 4**). However, the salt resistance of *ΔMA29250* was different from that of *ΔrhcQ*. The NaCl and LiCl tolerances of *ΔMA29250* were similar to those of the wild-type, while the KCl tolerance of *ΔMA29250* was decreased dramatically (**Figure 4**). Both mutants showed more sensitivity to KCl than to NaCl and LiCl, indicating that T3SS1 plays a more important role in resistance to KCl than to the other salts. All the complemented strains had similar salt resistance to the wild-type (**Figure 4**).

### Total Cellular Na^+^ and K^+^ Concentration Measurement

**FIGURE 5:**
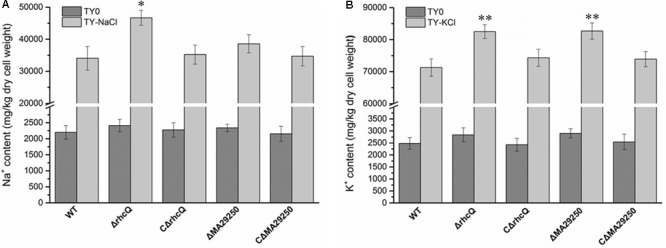
Total cellular Na^+^ and K^+^ concentrations of *M. alhagi* CCNWXJ12-2^T^. Wild-type (WT), mutants (*ΔrhcQ* and *ΔMA29250*), and the complementation strains (C*ΔrhcQ* and C*ΔMA29250*) were grown to OD_600_ ≈0.8 in TY broth containing appropriate antibiotics. A total of 500 microliters of each inoculum were plated on TY agar plates, TY plates containing 0.4 M NaCl, and TY plates containing 0.3 M KCl (all containing appropriate antibiotics) and inoculated at 28°C for 5 days. Then, the bacterial cells were collected for Na^+^ and K^+^ concentration measurement; those from TY plates containing 0.4 M NaCl were collected for Na^+^ concentration measurement, and those from TY plates containing 0.3 M KCl were collected for K^+^ concentration measurement. **(A)** Na^+^ concentrations in different strains; **(B)** K^+^ concentrations in different strains. Three independent experiments were conducted and the error bars indicate SDs. Significant differences between the WT strain and other strains are indicated (^∗^*P* < 0.05; ^∗∗^*P* < 0.01).

To explore the reasons for the decrease in salt resistance of the mutants *ΔrhcQ* and *ΔMA29250*, total cellular Na^+^ and K^+^ were measured after growth in 0.4 M NaCl and 0.3 M KCl, respectively. The Na^+^ and K^+^ concentrations in both mutants increased compared to the wild-type strain (**Figure 5**). In 0.4 M NaCl, the Na^+^ concentration in *ΔrhcQ* increased significantly (by about 37%) compared to the wild-type strain, while the Na^+^ concentration of *ΔMA29250* increased non-significantly (about 13%) (**Figure 5A**). In 0.3 M KCl, the increases in K^+^ concentration in strains *ΔrhcQ* and *ΔMA29250* were significant, both around 16% compared to the wild-type (**Figure 5B**). Although the increments of K^+^ concentrations in the mutants were lower than that of the Na^+^ concentration in strain *ΔrhcQ*, the overall K^+^ concentrations in all strains grown in 0.3 M KCl were about twice the Na^+^ concentrations in cells grown in 0.4 M NaCl (**Figure 5**). The higher K^+^ concentrations may explain the more serious growth repression caused by KCl than by NaCl shown in **Figure 2**. The Na^+^ and K^+^ concentrations in the complemented strains were similar to those in the wild-type.

### Effect of T3SS1 on Antioxidant Capacity in *M. alhagi* CCNWXJ12-2^T^

**FIGURE 6:**
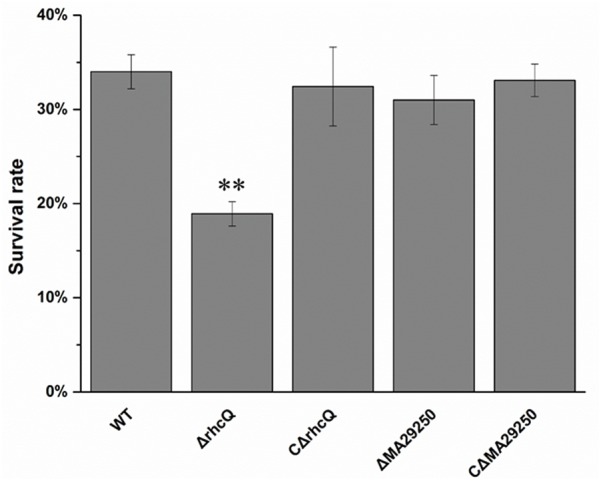
Survival rate of *M. alhagi* CCNWXJ12-2^T^ under oxidative stress. Wild-type (WT), mutants (*ΔrhcQ* and *ΔMA29250*), and the complemented strains (C*ΔrhcQ* and C*ΔMA29250*) were grown to OD_600_ ≈0.8 in TY broth containing appropriate antibiotics, and then adjusted to OD_600_ ≈0.2. The cultures were treated with 10 mM H_2_O_2_ for 30 min or incubated without H_2_O_2_ as a control. Cell survival rates were then counted and compared to untreated cells. The mean values of three independent experiments are shown and the error bars indicate SDs. Significant differences between the WT strain and other strains are indicated (^∗∗^*P* < 0.01).

**FIGURE 7:**
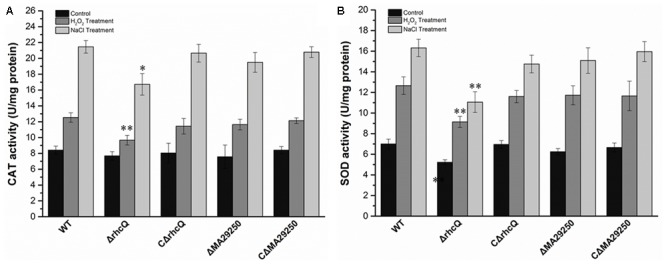
Antioxidant enzyme activities of *M. alhagi* CCNWXJ12-2^T^ under oxidative and salt stress. Catalase (CAT) and superoxide dismutase (SOD) activities of the wild-type (WT), mutants (*ΔrhcQ* and *ΔMA29250*), and complemented strains (C*ΔrhcQ* and C*ΔMA29250*) were measured in three independent biological experiments. **(A)** CAT activities; **(B)** SOD activities. Three biological replicates were conducted and the error bars indicate SDs. Significant differences of the enzyme activities in different conditions between the WT strain and other strains are indicated (^∗^*P* < 0.05; ^∗∗^*P* < 0.01).

To test whether T3SS1 has a role in oxidative stress resistance, we tested the antioxidant capacity of the mutants. The survival rate of *ΔrhcQ* after treatment with 10 mM H_2_O_2_ for 30 min was significantly decreased (by 50%) compared to the wild-type strain, while the survival rate of *ΔMA29250* was almost the same as that of the wild-type (**Figure 6**). The survival rates of the complemented strains after H_2_O_2_ treatment were also almost the same as that of the wild-type strain (**Figure 6**). The antioxidant enzyme activities of all the strains were measured in different conditions. In normal conditions (with no stresses), the SOD activity of *ΔrhcQ* showed a significant decrease, while the CAT activities of both mutants and the SOD activity of *ΔMA29250* showed no significant difference compared to the wild-type. The CAT and SOD activities of all strains were increased to varying degrees under oxidative stress (25–89%) and salt stress (110–160%) compared to normal conditions. The CAT and SOD activities of *ΔrhcQ* under salt and oxidative stresses were significantly decreased compared to the wild-type (68–78% of the level for the wild-type strain), while in *ΔMA29250*, the CAT and SOD activities showed no significant differences compared to the wild-type (**Figure 7**).

## Discussion

Our previous transcriptome analysis of *M. alhagi* CCNWXJ12-2^T^ in salt-free and high-salt conditions revealed different expression patterns of the T3SSs: T3SS1 was upregulated in high-salt conditions, while the expression of T3SS2 was downregulated (Liu et al., 2014). In the present study, phylogenetic analysis of the two T3SS core proteins, showed that T3SS1 and T3SS2 were members of subgroups α-Rhc II and α-Rhc I, respectively (**Figure 1**). Most T3SSs in subgroup α-Rhc I play important roles in symbiosis between the rhizobia and leguminous plants, while studies of the functions of T3SSs in subgroup α-Rhc II remain limited (Tampakaki, 2014). A former study showed that the expression of T3SS-II (belonging to α-Rhc II subgroup) in *Sinorhizobium* sp. NGR234 was upregulated in nodules formed with *Vigna unguiculata* and *Leucaena leucocephala* while the T3SS-II deletion mutant did not show any effect on nodule formation (Li et al., 2013). And another study of *P. aeruginosa* found that the osmaotic stress could induce the expression of T3SS with associated cytotoxins (Aspedon et al., 2006). In a study of *S. enterica* Serovar Typhimurium found that some simple salts, such as NaCl, KCl, NH4Cl, etc., could induce the assembly of SPI1-encoded T3SS and proteins secreted by T3SS (Mizusaki et al., 2008). Although the upregulation of the T3SS expression by simple salts was confirmed in these strains, the effects of T3SS on the salt tolerance were not studied. Here, we focused on the function of T3SS1, which is in subgroup α-Rhc II.

To explore the functions of T3SS1 in stress adaption by *M. alhagi* CCNWXJ12-2^T^, we first studied the promoter activities of T3SS1 on exposure to different salts and salt concentrations. β-galactosidase activities were used to reflect the promoter activities and increased with increasing concentrations of salts (**Figure 3**). This result encouraged us to study further the function of T3SS1 in *M. alhagi* CCNWXJ12-2^T^. Two genes were chosen to construct T3SS1-related mutants: *rhcQ* and *MA29250*. The loss of *rhcQ* could completely inhibit the assembly of T3SS in other bacteria (Biemans-Oldehinkel et al., 2011; Bzymek et al., 2012). Here, we hypothesized that the function of T3SS1 was completely inhibited in the *ΔrhcQ* mutant. The hypothetical protein encoding gene *MA29250* was randomly chosen from among the hypothetical proteins in the T3SS1 gene cluster for primary tests of its function. Our results showed a difference in stress resistance of the two mutants. Strain *ΔrhcQ* showed more sensitivity to NaCl, LiCl, KCl, and H_2_O_2_; *ΔMA29250* showed similar stress resistance to the wild-type strain, except toward KCl (**Figures 4, 6**). The results confirmed our hypothesis that the deletion of *ΔrhcQ* could completely disrupt the function of T3SS1; however, the deletion of *MA29250* only partially influenced the function of T3SS1. This phenomenon promoted us to study the role of T3SS1 in resistance to KCl.

The K^+^ concentration in both mutants was increased compared to the wild-type (**Figure 5B**), indicating that T3SS1 plays a role in adjusting the intracellular K^+^ concentration. Meanwhile, the Na^+^ concentration in both mutants was also higher than that in the wild-type strain, but there was a smaller increase in mutant *ΔMA29250* compared to *ΔrhcQ* (**Figure 5A**). These findings suggested that *rhcQ* plays a more important role in T3SS1 than *MA29250*, and that T3SS1 plays an important role in salt stress-resistance.

Environmental stresses such as hyperosmolality, acid, and high-salt concentrations can trigger secondary oxidative stress in bacteria (Mols and Abee, 2011). Levels of reactive oxygen species can be increased significantly when cells are grown in high-salt conditions (Koh et al., 2010; Calderon-Torres et al., 2011). Therefore, the oxidative stress resistance of the two mutants was measured. *ΔrhcQ* showed lower oxidative stress resistance than *ΔMA29250* and the wild-type (**Figure 6**). This result again suggested that *rhcQ* plays a more important role in T3SS1 than *MA29250*. CAT and SOD activities in *ΔrhcQ* were lower than those in the wild-type and *ΔMA29250* (**Figure 7**). Our results also showed that T3SS1 plays an important role in resistance to both salt and oxidative stress.

## Author Contributions

XL, ZL, and GW made conceived and designed of the study. XL and YL conducted the laboratory work. XL and ZL carried out the data analysis and manuscript writing. All the authors read and approved the final manuscript.

## Conflict of Interest Statement

The authors declare that the research was conducted in the absence of any commercial or financial relationships that could be construed as a potential conflict of interest.

## Footnotes

1 https://www.ncbi.nlm.nih.gov/
