# Supplementary material for: Functional Analysis of a Putative Type III Secretion System in Stress Adaption by Mesorhizobium alhagi CCNWXJ12-2T
Source: Front Microbiol. 2018 Feb 20;9:263. doi: 10.3389/fmicb.2018.00263 (PMC5826200; doi:10.3389/fmicb.2018.00263)
Supplement: Supplementary file 1 [file Table_1.DOCX]

*Supplementary Material*

**Functional Analysis of the Type III Secretion System in Stress Adaption by *Mesorhizobium alhagi* CCNWXJ12-2^T^**

**Xiaodong Liu^1^, Yantao Luo^1^, Zhefei Li^1*^, Gehong Wei^1*^**

^1^ College of Life Sciences, State Key Laboratory of Crop Stress Biology in Arid Areas, Northwest Agriculture & Forestry University, Yangling, Shaanxi 712100, China

*** Correspondence:**

Gehong Wei

[weigehong@nwsuaf.edu.cn](mailto:weigehong@nwsuaf.edu.cn)

Zhefei Li

lizhefei@hotmail.com

**Table S1** Primers used in this study.

| Primer | Sequence^a^ (5'-3') | Note |
| --- | --- | --- |
| TP1 | CCCAAGCTTCTTTTCGAATTCCGTACTGGC | To generate promoter reporter plasmid pBMLTP13 |
| TP3 | GCTCTAGACTACTGCGTGGCGACCGGCG |  |
|  |  |  |
| RhcQ-US | GGAATTCCTTGCCGAGACGCTGCGG | To generate gene knockout plasmid pK18*rhcQ* |
| RhcQ-UA | GCTCTAGAATGCGTCGACTTCCGGCTGCG |  |
| RhcQ-DS | GCTCTAGAGCATTCGCGTCAGGCGCATTT |  |
| RhcQ-DA | CCCAAGCTTGCCAAGCGTCATCAGCACATT |  |
|  |  |  |
| MA29250-US | GGAATTCCTTCAGCGCCGAATCGGC | To generate gene knockout plasmid pK18*MA29250* |
| MA29250-UA | GCTCTAGAGCTCACCGCATTGCGCGT |  |
| MA29250-DS | GCTCTAGACCTTGGCAAGGACGGGGC |  |
| MA29250-DA | CCCAAGCTTGGTGGTTGTCGAGGAACC |  |
|  |  |  |
| CRhcQA | GTCGCCACGCAGTAGGCTCTAGAGATGCTGAAATCCGCATCCTCTCCCC | To generate complement plasmid pBML*rhcQ* |
| CRhcQB | GTGAAATTGTTATCCGCTCTAGATCATTTTGCGAAAATGCGCCTGACG |  |
|  |  |  |
| CMA29250A | GTCGCCACGCAGTAGGCTCTAGAGATGGGCGGCGAACCGGGCCTC | To generate complement plasmid pBML*MA29250* |
| CMA29250B | GTGAAATTGTTATCCGCTCTAGATCATGGGTACTTCAGGGAAATG |  |

^a^Restriction enzyme sites and homologous sequences are underlined.
